# Recognizing early signs in the translational phase is essential for drug development in cardiovascular medicine

**DOI:** 10.1002/ehf2.14402

**Published:** 2023-05-10

**Authors:** Zoltán Papp, Piero Pollesello, Attila Borbély

**Affiliations:** ^1^ Department of Cardiology, Division of Clinical Physiology, Faculty of Medicine University of Debrecen Debrecen Hungary; ^2^ Content and Communications Function, Orion Pharma Espoo Finland; ^3^ Department of Cardiology, Faculty of Medicine University of Debrecen Debrecen Hungary

We have just returned from a very inspiring Winter Meeting on Translational Heart Failure Research, organized by the Heart Failure Association of the European Society of Cardiology (ESC) in collaboration with the ESC Working Group of Myocardial Function. In keeping with the traditions of this meeting there was a strong focus of enabling young researchers to confer with a faculty of seasoned colleagues about translational aspects of heart failure therapy development.

The first session of the 2023 meeting was dedicated to ‘How to avoid translational failures’ and included a lecture on why and how to ‘see early signs in the translational phase to avoid subsequent problems in the clinical stage’. This is an extremely important subject not only to avoid loss of time and the misallocation of funds and resources, but foremost to avoid patients entering clinical phases that are destined to fail or, conversely, to reduce the risk that promising new therapies will be overlooked.

In assessing these early signs it is important to take a wide view of the situation—information acquired in the preclinical phase for a new chemical entity or a novel method may provide valuable, even essential, information during the development of different chemical entities or a related method. This principle extends to ‘negative’ findings such as warning signs linked to the chosen molecular target itself, or to prodrug construction. Ignoring early signals is often costly and can become dangerous when candidate therapies move into clinical evaluation.

The recent history of cardiovascular research is not lacking in examples of these pitfalls.

1. At the start of the 21st century there was considerable enthusiasm and high hopes for the rapid advancement of cell therapies for the management of postischaemic cardiomyopathy. This anticipation was grounded in preclinical findings which challenged an established consensus that asserted the impossibility of continuous myocyte regeneration in the human heart.^1^, ^2^ These revolutionary concepts caught public imagination and stimulated the transfer of cell therapies from the bench to the bedside. Numerous clinical trials using various stem cell types on large number of patients with acute myocardial infarction were tested worldwide.^3^ There was perhaps already a warning, however, in commentaries that alluded feasibility being demonstrated in ‘small, mostly uncontrolled trials.’^3^ Full evaluation of the lasting effects of these complicated and expensive treatments in large randomized controlled trials and meta‐analysis let much of the air out of the balloon^4^, ^5^ and provide an example of why special caution is required when extrapolating from early results in preclinical studies to the hearts of our patients.
2. More recent experience relates to omecamtiv mecarbil (a direct myosin activator) where it appears that during the development of this drug an early discussion on sarcomere‐active drugs was, to borrow a phrase, ‘lost in translation’. In the years preceding the emergence of omecamtiv mecarbil various molecules now referred to as myosin activators were seen to prolong the contractility transient and were abandoned because they were deemed potentially harmful in ischaemic conditions.^6^, ^7^, ^8^ Unfortunately, omecamtiv mecarbil shares some of these characteristics, and in one of its early clinical trials an increased propensity for myocardial ischaemia was implicated, albeit at relatively high drug concentrations.^9^, ^10^ That might have been a chance observation, but it might equally have been related to signs seen in the translational phase and to a signal of cardiac ischaemia noted in a Phase II trial.^11^ On 1 March 2023, the oral formulation of omecamtiv mecarbil was not granted authorization by the Food and Drug Administration on the grounds of lack of evidence on efficacy despite a vast clinical program including a total of over 10 000 patients in 11 investigational and regulatory clinical trials even though drug concentrations were carefully kept at minimum levels.
3. As an example in which positive signs were ignored or went unappreciated for longer than necessary we cite the case of the sodium‐glucose cotransporter 2 (SGLT2) inhibitors. The first review on SGLT2 as a therapeutic target was published in 2010 but a further 7 years elapsed before the potential of SGLT2 inhibitors for heart failure beyond diabetes was acknowledged in print.^12^, ^13^ The early positive signs of the added value of these antidiabetic agents for heart failure took time to be fully understood and exploited.

One can readily recognize the consequences in all three examples. On one hand, time and resources lost and patients enrolled in fruitless trials, on the other, patients waiting too long for a successful treatment to be introduced in clinical use. All these outcomes are clear evidence of limitations in the methodology of drug development. A separate but connected consideration is that, from an industrial perspective, failures in drug development may contribute to funds being diverted to other areas seen as more lucrative, with consequences for future research in what may be seen as ‘niche’ areas such as e.g. acute heart failure.

The central lesson we take from these examples is the necessity of continuous and open scientific discussions among independent researchers, trialists, representatives of the pharma‐industry and regulatory authorities. Moreover, while the significance of the step‐by‐step approach for cardiovascular drug development cannot be underestimated, every effort should be made to avoid siloed activity and thinking by all of these participants.

In the current complicated business environment, we are grateful to companies that still support innovative research and expensive clinical developments. We also know that to attract and retain the support of investors it is crucial for such companies to create expectations. Acute, chronic, and advanced heart failure are, however, not simple therapeutic development arenas,^14^ and it would be both morally and commercially wrong to encourage overoptimistic expectations.^15^ Close and rigorous scrutiny of early signs in the translational phase is crucial to avoid mistakes and to optimize the development of new therapeutics.

## Conflict of interest

Zoltán Papp and Attila Borbély have nothing to declare. Piero Pollesello is full time employee at Orion Pharma.
